# A grapeful discovery: Reservoir computing with wine beads

**DOI:** 10.1093/pnasnexus/pgaf335

**Published:** 2025-10-22

**Authors:** Raphael Fortulan, Noushin Raeisi Kheirabadi, Alessandro Chiolerio, Andrew Adamatzky

**Affiliations:** Centre for Sustainable Computing, University of Huddersfield, Queensgate, Huddersfield HD1 3DH, United Kingdom; Unconventional Computing Laboratory, University of the West of England, Coldharbour Ln, Stoke Gifford, Bristol BS16 1QY, United Kingdom; Unconventional Computing Laboratory, University of the West of England, Coldharbour Ln, Stoke Gifford, Bristol BS16 1QY, United Kingdom; Unconventional Computing Laboratory, University of the West of England, Coldharbour Ln, Stoke Gifford, Bristol BS16 1QY, United Kingdom; Bioinspired Soft Robotics, Istituto Italiano di Tecnologia, Via Morego 30, Genova, GE 16163, Italy; Unconventional Computing Laboratory, University of the West of England, Coldharbour Ln, Stoke Gifford, Bristol BS16 1QY, United Kingdom

## Abstract

In this work, we introduce encapsulated wine beads as a novel, edible material for unconventional and neuromorphic computing. When encapsulated in alginate beads, wine, a complex mixture of proteins, organic acids, sugars, metal ions, and volatiles, exhibits nonlinear electrical behavior and memory effects governed by ox–redox processes. These responses show plasticity-like features, allowing programmable resistance states. The wine beads can be leveraged for computing, and to demonstrate this potential, the wine bead was used as a single-node reservoir for classification tasks. Our findings indicate that the resistance is programmable, exhibits a high degree of repeatability, and can be used for reservoir computing scenarios.

Significance Statement This research demonstrates the potential of edible materials for unconventional computing applications. By leveraging the unique electrical properties of wine, we have shown that wine can be used for physical reservoir computing. This work opens up new possibilities for sustainable and biodegradable materials impacting food science, biotechnology, and environmental engineering.

## Introduction

Traditional ingestible electronics face challenges with their nondegradable components and complex retrieval. Edible electronics offer an innovative approach by using fully biodegradable material (1). These developments align with sustainable engineering and chemistry to minimize environmental impact while enhancing device efficiency and safety (2–4).

In biomedicine, gastrointestinal devices perform diagnostics, monitor health, and deliver drugs without hospitalization (5, 6). The food industry also benefits from real-time product monitoring, safety, and traceability, potentially reducing food waste (7, 8). This convergence addresses the challenges of personalized medicine and sustainable food management. In this work, we envision the application of edible materials/electronics to unconventional computing (UC), exploring alternative substrates for computational tasks (9).

While UC encompasses diverse substrates, from DNA computing (10, 11) and quantum computing (12) to collision-based computing using *Mictyris guinotae* soldier crabs (13), bio-based solutions have demonstrated promising computational capabilities. For example, fungal mycelia have been used to control robots (14) and fungal colonies can be used as electrical gates (15). More recently, colloidal substrates of synthetic origin have also been shown to be capable of possessing basic features such as memory (16), Pavlovian learning (17), Boolean gates (18), and other computing capabilities (19).

Recent advancements in UC have also increasingly focused on neuromorphic computing, which aims to design hardware and algorithms that mimic the structure and functionality of biological neural networks for efficient, brain-inspired information processing (20–22). These systems integrate memory and computation within the same physical structure, reducing energy consumption, and improving processing efficiency.

Among neuromorphic computing technologies, resistive random-access memory (ReRAM)-based memristors have gained significant attention due to their small footprint, low power consumption, and ability to exhibit synaptic-like behavior (23, 24). Memristive crossbar arrays have demonstrated key neuromorphic functionalities, including unsupervised pattern classification, multilayer perceptron networks, and efficient differential equation solving (25, 26).

In this study, we explore wine as a substrate for UC. Wine’s historical significance spans millennia, deeply woven with human civilization, probably first produced in the Caucasian area, spanning from ancient Egyptian and Greek civilizations through the Roman Empire, valued for its cultural, social, and religious importance. Its profound impact is perhaps best captured by Petronius’s famous quote “*vinum vita est*” (wine is life) (27). In addition to its historical significance, wine has unique electrical properties. It contains a rich composition of cations (Na^+^, K^+^, Ca^2+^, Mg^2+^, and NH^4+^), organic anions from multiple acids (malic, citric, fumaric, tartaric, oxalic, and formic), and inorganic anions such as SO_4_^2-^ and PO_4_^3-^ (28–31).

Wine was enclosed in an edible gel-like shell using alginate, a naturally occurring biopolymer derived from brown sea algae (32). Its properties, such as biocompatibility, biodegradability, nontoxicity, and nonimmunogenicity, render it a nearly ideal and long-sought-after material for use in edible electronics (33–35). This success is derived from the ability to create an electrostatic bond between these polymer chains with divalent ions such as Ca^2+^, Sr^2+^, and V^2+^ and form hydrogels (36).

This work shows that wine encapsulated in an alginate hydrogel exhibits nonlinear electrical behavior, memory effects, and resistance switching, features that closely mimic the adaptive properties of biological synapses. We further demonstrate that this biomaterial can be used for physical reservoir computing (37, 38).

## Materials and methods

### Wine production

The wine used in this work is a red fruity variety called “Barbera,” elaborated using traditional methods that preserve natural ferments in the harvest season 2024 (cantina Adorno, Vigliano d’Asti, Monferrato, Italy). Unfiltered must was shipped to the United Kingdom, where fermentation was completed in our laboratory before any tests. After fermentation, the unfiltered wine was aliquoted and stored at 5°C until use. Wine density, pH, and ethanol content were 0.991 gmL${\;}^{-1}$, 3.02, and 12.01%, respectively.

### Wine hydrogel beads preparation

Wine beads were synthesized by creating a homogeneous mixture of 1 g of sodium alginate (NaC_6_H_7_O_6_, Merck) dissolved in 100 mL of deionized water under stirring at 700 rpm and 50°C for 30 min. In sequence, 100 mL wine was added to the solution.

Finally, the mixture was added dropwise to 500 mL of an aqueous calcium chloride (CaCl^2^, Merck) solution (3 wt%) at room temperature (39). The resulting beads were washed with deionized water and kept inside the wine to prevent the solvent exchange.

**Fig. 1.**
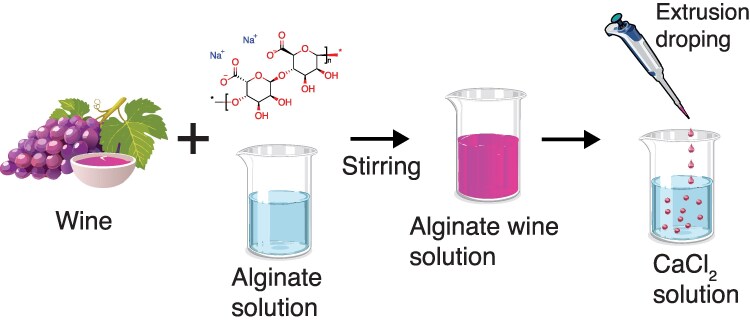
Description of the experimental procedure used to synthesize wine beads.

The experimental setup for the preparation of the wine beads is illustrated in Fig. 1.

### Electrical measurements

The current and voltage characteristics of the wine bead were measured using a USB-6421 mioDAQ data acquisition device (National Instruments, USA) connected to a tinyCurrent shunt and amplifier (n-fuse GmbH, Germany). The device was controlled via the NI-DAQmx Python API, and input sine waves were generated using the internal DAQ system.

For pulse application, a Pulse Pal v2 pulse train generator (Sanworks, USA) was used to deliver the stimulus sequence to the wine bead. The generator was controlled in parallel with the mioDAQ system using the Pulse Pal Python API.

All measurements were conducted using Pt/Ir-coated stainless-steel probes (10 μm diameter, Spes Medica, Italy), which were inserted into the beads to establish electricalconnections. To reduce measurement noise, the acquired data were filtered using LOWESS with a windowsize of 450 points.

### Computational

The Iris dataset, which comprises four features (40) comprises four features (petal length, petal width, sepal length, and sepal width) of three Iris flower species and was employed to evaluate the memory and programming capabilities of the wine bead for reservoir computing.

Each feature was individually scaled to a range of ±1 and then linearly mapped to ±2.5V, 50 ms voltage pulses. For each example, the four features were applied sequentially, followed by a final 0.5 V, 50 ms pulse to read the resistance state of the material. At the end of each example, a -5 V, 50 ms pulse was applied to reset the reservoir’s resistance state.

The dataset was partitioned into training and testing sets using a stratified 70%/30% split, with 5-fold cross-validation performed to assess model performance. Data processing and evaluation were conducted using Python 3.11.5 on an ARM-based CPU (M2 Max, Apple), and the output layer was trained using scikit-learn 1.5.2.

## Results

**Fig. 2.**
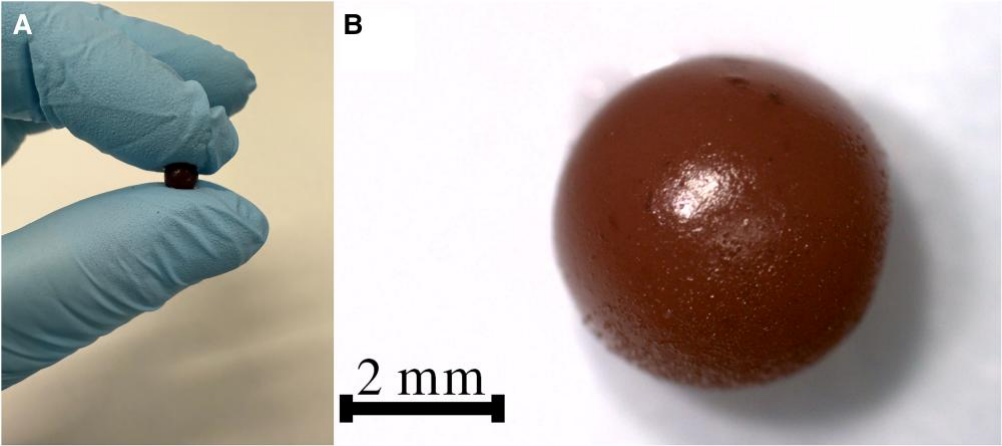
A) Photograph and B) optical microscopy image of a prepared wine bead.

The synthesized wine beads, shown in Fig. 2A and B, have an average radius of ∼2.2 mm and a mass of ∼5.5 mg. Optical microscopy confirms that the beads are uniform in size and morphology.

### Electrical characterization

**Fig. 3.**
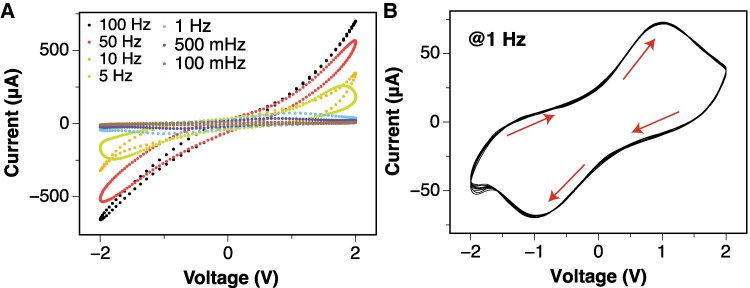
A) Averaged *I–V* characteristics of the wine bead under a 2 V peak sine wave at different frequencies. B) Evolution of the *I–V* response over 10 cycles at 1 Hz.

Current–voltage (*I–V*) measurements were performed using two Pt/Ir electrodes inserted into the wine bead. Figure 3A shows the averaged *I–V* characteristics recorded over 10 cycles under an AC voltage sweep with a 2 V peak sine wave at frequencies ranging from 100 mHz to 100 Hz.

The presence of hysteresis loops in these curves indicates memory effects (41–43). The nonlinear dependence of the current on the input voltage also tends to collapse as the input frequency increases, which is a characteristic of memfractive systems (44–46).

Figure 3B shows the evolution of the *I–V* response at 1 Hz over 10 cycles. Under a positive bias, the bead transitions from a low resistance (LRS) to a high resistance (HRS). The application of a negative bias reverses this transition.

The primary oxidizable substances in wine are polyphenols, such as (+)-catechin, which oxidizes to quinone, and ethanol, which oxidizes to acetaldehyde (47). When the platinum electrodes are immersed in aqueous wine and the applied potential is progressively positively increased, an anodic current is generated due to the oxidation of ethanol to acetaldehyde, followed by the oxidation of water to oxygen.

At higher positive potentials, electrons are extracted from polyphenols, leading to an anodic current that increases to a peak (as shown in Fig. 3B) when most of the polyphenol near the electrodes is oxidized to quinone (48). During the reverse potential sweep, a cathodic current is observed as quinone is reduced back to catechol, producing a cathodic peak when most of the quinone has been reduced. The transport of polyphenols and quinones to and from the electrode is governed by diffusion, which determines the observed current peaks and the overall shape of the voltammogram.

### Resistance modulation

**Fig. 4.**
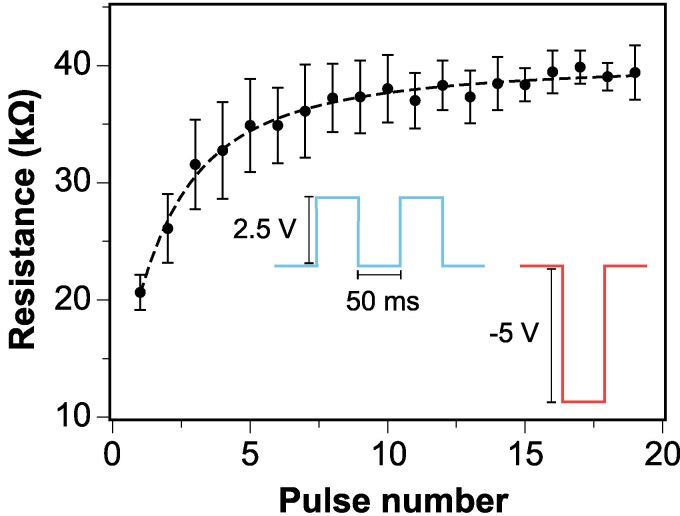
Measured resistance values for 19 set pulses (2.5 mV, 50 ms) followed by one -5 V reset pulse over 20 repetitions. Median values are shown with interquartile range error bars.

To assess the controllability of the resistance states of the material, we applied a sequence of 19 set pulses (2.5 V, 50 ms), followed by a single -5 V, 50 ms reset pulse, repeated over 20 cycles. As shown in Fig. 4, the resistance values exhibit consistent, reproducible transitions between well-defined states. This behavior driven is by redox reactions and ionic migration within the wine-alginate matrix.

The ability to modulate resistance states is essential for neuromorphic functionality, as it enables information encoding and storage in a manner analogous to synaptic weight adjustments in biological systems. This resistive switching process parallels key memory functions, such as short-term memory (STM) and short-term depression (STD), observed in biological synapses (49, 50). The transition from a low resistance state (with high conductivity) to a high resistance state (lower conductivity) using the input pulses mimics STD, where synaptic conductances (synaptic strength) decrease over the application of stimuli (51). Conversely, the recovery after the reading pulse from HRS to LRS represents memory retention and recall, akin to STM information holding in biological synapses.

As mentioned early, the neuromorphic performance of the wine beads is directly linked to the ionic dynamics within the liquid material. The electrical characteristics of the beads are influenced by the complex chemical composition of red wine, which contains various metal-ion ligands—such as polysaccharides, peptides, proteins, and polyphenols—as well as metal ions (52, 53). The metal content of wine evolves significantly during fermentation, maturation, and storage (54), affecting its electrical properties.

Fermented wines generally exhibit lower metal ion concentrations than grape juice or must due to precipitation of tartrates and formation of insoluble metal complexes. Changes in pH and alcohol content during fermentation further accelerate this process, while yeast absorption of metals like Ca, Cu, Fe, K, Mg, and Zn contributes to concentration shifts.

Despite these reductions, some wines, including the Italian variety used in this study, retain elevated levels of metal ions such as Ba, Ca, Co, Cr, Li, Rb, Sr, V, and Mg (55) and likely play a crucial role in defining the electrical properties of the wine bead, influencing its resistive switching behavior.

### Example application: reservoir computing

To illustrate a possible application of the wine-alginate bead, we employed the wine bead as a reservoir layer for classification tasks within a physical reservoir computing framework (56). In physical reservoir computing, the reservoir is a dynamic physical system that nonlinearly transforms input signals and temporally encodes them into a higher-dimensional space (57).

**Fig. 5.**
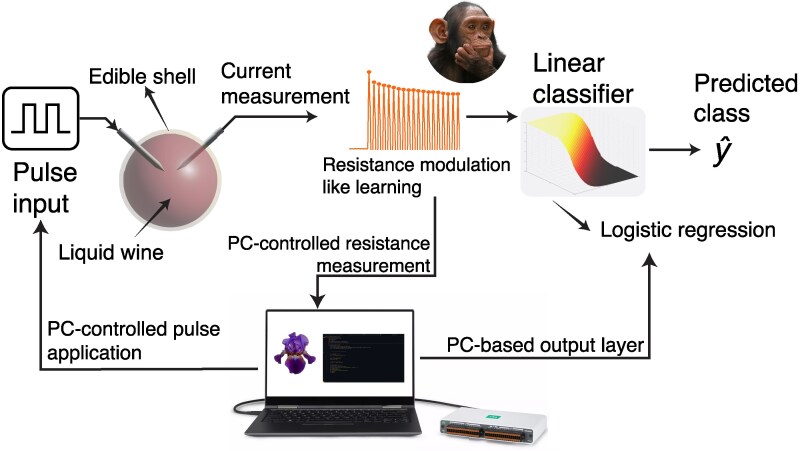
Experimental setup used for the reservoir computing using the wine bead. Photo credit: Vladimir Wrangel.

In our implementation, the reservoir is a single-node system, requiring 1D inputs and outputs. So to evaluate the performance of the wine bead reservoir, the Iris dataset was converted into a series of input voltage pulses, as described in the Materials and methods section. We explored the neuromorphic characteristics in the wine bead, as depicted in Figs. 4 and 5, where synaptic behavior was modulated by the timing and polarity of applied pulses.

Positive pulses delivered in rapid succession increased the resistance, mimicking paired-pulse facilitation observed in biological neurons (58), whereas negative pulses decreased the resistance, resembling paired-pulse depression of biological synapses (59).

**Fig. 6.**
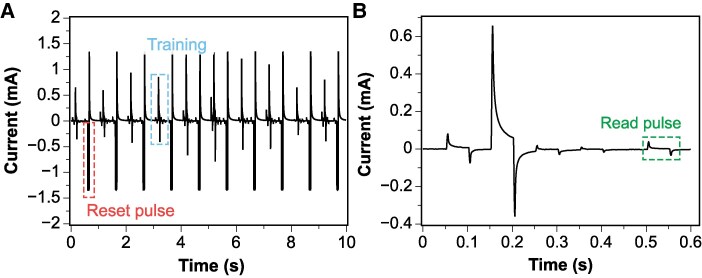
A) Current response during the initial 10 s of the training phase, illustrating resistance modulation during set and reset pulses. B) Zoomed-in view of the read pulse in the first 600 ms.

Figure 6A illustrates the current response during the training phase, showing resistance set and reset cycles, while Fig. 6B provides a zoomed-in view highlighting the read pulse. The final state of the reservoir (resistance value measured during the read pulse) was then used as input for logistic regression.

The overall accuracy of the physical reservoir scheme was 93%, slightly outperforming the accuracy obtained when applying the data directly to logistic regression. However, these results demonstrate that the nonlinear dynamic response of the state-switching resistance can be exploited as a physical reservoir. Moreover, the temporal multiplexing of the reservoir, which requires sufficiently low cycle-to-cycle variability for reliable operation, was successfully achieved.

## Conclusion

This study explored the properties of wine encapsulated in an alginate hydrogel for reservoir computing applications. The material showed nonlinear electrical responses and the ability to switch between resistance states, which are relevant characteristics for reservoir computing approaches. Using the Iris dataset, we demonstrated that the wine-alginate system can be used in reservoir computing as a single-node reservoir by modulating its electrical resistance through applied voltage pulses.

The electrical characteristics of the wine-alginate system are linked to its complex chemical composition. The material’s behavior is influenced by a rich mixture of metal–ion ligands, including polysaccharides, peptides, proteins, and polyphenols. These compounds play a crucial role in defining the rich and unique electrical properties of the wine bead and demonstrate how complex biological systems can be leveraged for unconventional computing applications, bridging the domains of food science, materials engineering, and computational technology.

## Data Availability

Dataset generated in this work is available at 10.5281/zenodo.15586269.
